# VAR2CSA Ectodomain Labeling in *Plasmodium falciparum* Infected Red Blood Cells and Analysis via Flow Cytometry

**DOI:** 10.21769/BioProtoc.4725

**Published:** 2023-08-05

**Authors:** Olivia M.S. Carmo, Matthew W.A. Dixon

**Affiliations:** 1 Department of Biochemistry and Pharmacology, Bio21 Molecular Science and Biotechnology Institute, University of Melbourne, Parkville, Australia; 2 Department of Infectious Diseases, Peter Doherty Institute for Infection and Immunity, University of Melbourne, Melbourne, Australia; 3 Division of Infectious Diseases and Immune Defense, Walter and Eliza Hall Institute of Medical Research, Parkville, Australia

**Keywords:** Malaria, Flow cytometry, Protein transport, Surface proteins, Live cell

## Abstract

Presentation of the variant antigen *Plasmodium falciparum* erythrocyte membrane protein 1 (EMP1) at the surface of infected red blood cells (RBCs) underpins the malaria parasite’s pathogenicity. The transport of EMP1 to the RBC surface is facilitated by a parasite-derived trafficking system, in which over 500 parasite proteins are exported into the host cell cytoplasm. To understand how genetic ablation of selected exported proteins affects EMP1 transport, several EMP1 surface presentation assays have been developed, including: 1) trypsinization of surface-exposed EMP1 and analysis by SDS-PAGE and immunoblotting; and 2) infected RBC binding assays, to determine binding efficiency to immobilized ligand under physiological flow conditions. Here, we describe a third EMP1 surface presentation assay, where antibodies to the ectodomain of EMP1 and flow cytometry are used to quantify surface-exposed EMP1 in live cells. The advantages of this assay include higher throughput capacity and data better suited for robust quantitative analysis. This protocol can also be applied to other cellular contexts where an antibody can be developed for the ectodomain of the protein of interest.

## Background

The most virulent form of malaria is caused by the protozoan parasite *Plasmodium falciparum*, killing over 600,000 people annually (Weiss et al., 2019). During the asexual blood stage, the parasite invades the red blood cell (RBC) and feeds on intracellular hemoglobin; however, while circulating in the host bloodstream, the infected RBC is at risk of elimination if it transits the host’s splenic sinuses. To avoid splenic clearance, the parasite remodels the host cell by exporting proteins into the RBC cytoplasm (Marti et al., 2004; Sargeant et al., 2006; Boddey et al., 2013; Heiber et al., 2013). During this process, a key modification is the presentation of the variant antigen *P. falciparum* erythrocyte membrane protein 1 (EMP1) at the surface of infected RBCs (Smith et al., 1995). The antigen EMP1 is encoded by the *var* gene family where each parasite expressing one of approximately 60 variants at any time. These variants act as adhesins to a range of cellular ligands, expressed on a range of endothelial cells in the capillaries throughout the body, allowing the infected RBC to sequester within the host’s vasculature. The cytoadhesion of infected RBCs can lead to fatal complications associated with cerebral and placental malaria (Storm and Craig, 2014; Jensen et al., 2020; Sahu et al., 2021). EMP1 transport to the surface remains poorly understood as the parasite, with the parasite building its own *de novo* trafficking system, which is highly divergent from classical eukaryotic trafficking machinery. For these reasons, EMP1 trafficking is of high interest from both clinical and basic biology perspectives.

In our recent studies, we identified parasite proteins that are exported into the host cell and affect the trafficking and presentation of the antigen EMP1 (McHugh et al., 2020; Carmo et al., 2022). The majority of EMP1 is trafficked to the host cell surface 16–20 h post invasion, and mid-trophozoite stage-infected RBCs (20–32 h post invasion) are used to study EMP1 presentation at the host cell surface (Kriek et al., 2003). To determine if EMP1 is present at the surface of infected RBCs, we employ two complementary assays. These include: 1) trypsin cleavage assay, where surface-exposed EMP1 is shaved from the surface by trypsin and the membrane-embedded domain is then detected by immunoblotting (Cooke et al., 2006; Carmo et al., 2022); and 2) binding assays, in which the infected RBCs are passed through a channel coated with ligand and the number of cells adhering is quantified (McHugh et al., 2015 and 2020; Carmo et al., 2022). These assays are useful; however, they have their limitations. For example, the trypsin assay is semi-quantitative at best, while defects in adhesion underflow can be multifactorial (not due to EMP1 surface presentation alone).

**Figure 1.**
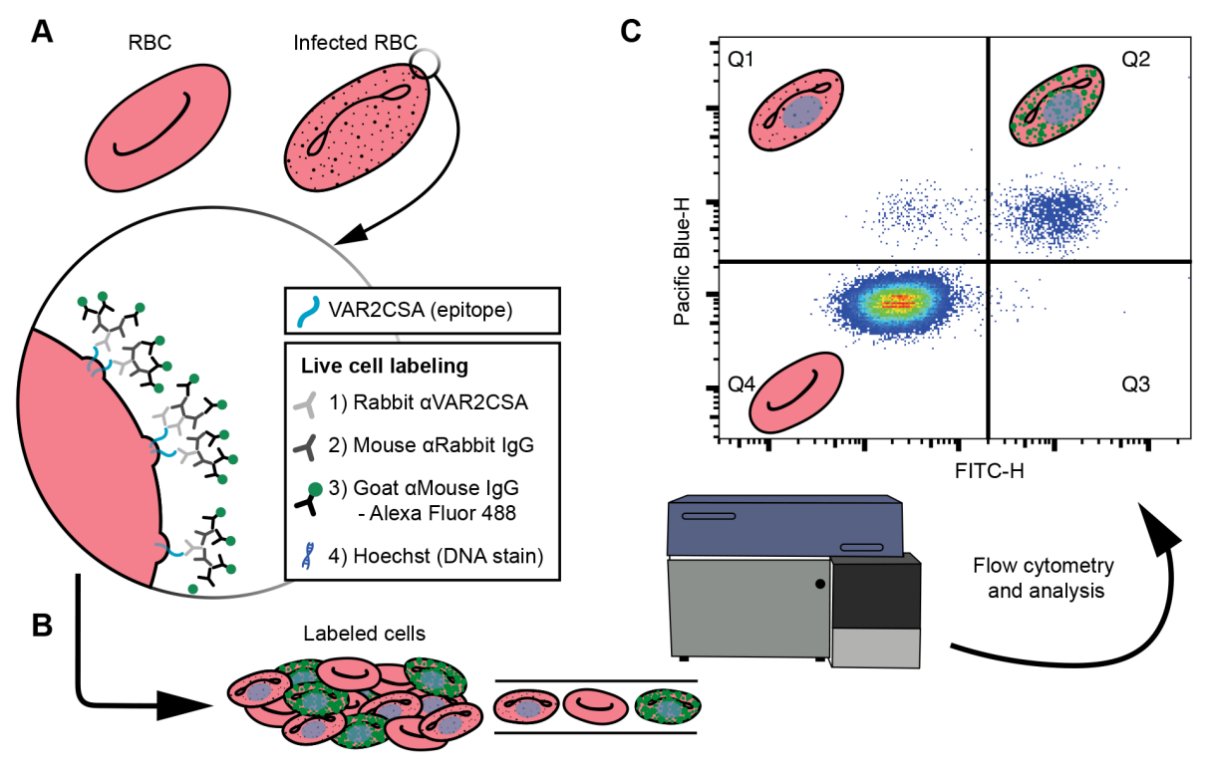
Cell labeling and analysis flowchart. (A) Live cell labeling of the ectodomain of the VAR2CSA, an EMP1 variant available on the surface of *P. falciparum* infected red blood cells (RBCs). The VAR2CSA variant is associated with adhesion to chondroitin sulphate A in the placenta, causing placental malaria. A zoom-in window depicts the VAR2CSA antigen (blue) with a list of the live cell labeling steps: incubations with three antisera, the final of which is conjugated to an Alexa Fluor 488, and finally a DNA stain. (B) The labeled cells are analyzed by flow cytometry. (C) Major populations can be separated by plotting the Pacific blue height (cells with DNA) against FITC height (cells labeled with Alexa Fluor 488), where each dot is a single cell and high densities of cells are indicated by blue through to red hues. Schematics of RBCs indicate the type of cell gated in each quartile. Uninfected RBCs do not have DNA; therefore, Q4 represents uninfected cells. Q1 represents infected cells that do not have VAR2CSA ectodomains available to the antisera. Q2 indicates infected cells that have VAR2CSA ectodomains available to the antisera. These data are used to compare the Q2 populations between cell lines to determine if mutations to *P. falciparum* cell lines affect VAR2CSA translocation to the surface of the infected RBC.

In an alternative approach outlined here, antibodies specific to the ectodomain of EMP1 are used to label live cells, which are then quantitated by flow cytometry, to measure the number of labeled cells and their relative intensity of surface-exposed EMP1 (first established in Smith et al., 1995; Beeson et al., 2004; Elliott et al., 2005) (Figure 1). Of the techniques assaying EMP1 surface presentation published to date, this is the most time- and resource-efficient method. In addition, the assay is implemented in a 96-well plate format, making it easily scalable. This technique can also be adjusted to quantitate surface presentation of surface proteins in other cellular contexts where an antibody is available for the ectodomain of the protein of interest.

## Materials and reagents

*Storage notes: All primary antibodies are stored at -20 °C. Secondary antibodies and DNA stains are stored at 4 °C unless otherwise noted. All reagents for tissue culture were prepared in a class II biosafety cabinet while practicing aseptic technique, including the use of sterile pipette tips*.

1. 96-well plate, v-well, polystyrene with lid, sterile (Sarstedt, catalog number: 3 82.1583.001)
2. Sterile 35 mm × 10 mm tissue culture dish (Sigma, Corning, catalog number: 430165)
3. BSA 30% (w/v) (CSL Immulab, catalog number: 06701305)
4. Rabbit anti-VAR2CSA antibody recognizing an epitope on the ectodomain of VAR2CSA [Duffy and Rogerson labs, University of Melbourne, code: R1945 (Reeder et al., 1999)]
5. Monoclonal anti-rabbit IgG (γ-chain specific) antibody produced in mouse (Sigma, catalog number: R1008-2ML)
6. Alexa Fluor 488 goat anti-mouse IgG (H+L) (Invitrogen, Life Technologies, catalog number: A11029)
7. Alexa Fluor 647 goat anti-mouse IgG (H+L) (Invitrogen, Life Technologies, catalog number: A21236)
8. Hoechst 33342 (Invitrogen, catalog number: H3570)
9. SYTO 61 (Life Technologies, catalog number: S11343) to measure parasitemia. SYTO 61 is diluted from 5 mM to 100 μM in DMSO and stored in 50 μL aliquots at -20 °C
10. Dimethyl sulfoxide (DMSO) (ChemSupply, catalog number: DA013-500M)
11. RPMI 1640 medium with GlutaMAX supplement and HEPES (Life Technologies, Gibco, catalog number: 72400120)
12. Hypoxanthine (Sigma, catalog number: H9636-5G) made to 200 mM in 1 M NaOH (ChemSupply, catalog number: SL178-500G), filter sterilized, and stored in 1 mL aliquots at -20 °C
13. Gentamicin 10 mg/mL in deionized water (Sigma, catalog number: G1397)
14. D-glucose (ChemSupply, catalog number: GA018-500G)
15. D-sorbitol (ChemSupply, catalog number: SL151-500G), made to 5% w/v in MilliQ water, filter sterilized, and stored at 4 °C
16. Albumax II (Life Technologies, catalog number: 11021045) dissolved in RPMI 1640 medium with GlutaMAX supplement and HEPES at 5%, filter sterilized, and stored in 25 mL aliquots at -20 °C
17. Media solution (910 mM D-glucose, 0.45 mg/mL gentamicin), stored in 5 mL aliquots at -20 °C
18. Pooled sera from any blood type (Lifeblood Australia), heat inactivated for 1.5 h, filter sterilized, and stored in 25 mL aliquots at -20 °C
19. Red blood cells (Lifeblood Australia), O+
20. Malaria mix (1% O_2_, 5% CO_2_, and 94% N_2_) (Coregas, catalog number: 388150)
21. Giemsa’s stain improved solution R66, Gurr for microscopical staining (VWR chemicals, catalog number: 350864X)
22. Methanol (ChemSupply, catalog number: MA004)
23. Triton X-100 (BioXtra, Sigma, catalog number: T9284)
24. Sodium hypochlorite 8-14% (Ajax Finechem, catalog number: AJA485-5L)
25. Complete culture media (CCM) stored at 4 °C, used at 37 °C (see Recipes)

## Equipment

1. Incubator (LabQuip Sciences, SEM Equipment, model: 18FD)
2. Multichannel pipettes, 8-channel, 20–200 μL (Socorex, model: 855) and 5–100 µL (Bohit, model: m100)
3. Class II biosafety cabinet (Laftech, EuroClone, model: safemate 1.2 vision)
4. Centrifuge (Hettich, model: Rotina 420)
5. Flow cytometer [BD Biosciences, model: FACSCanto II Flow Cytometer System with an integrated BD High Throughput Sampler (HTS)]. The following filter sets were used for fluorophore detection: Alexa Fluor 647 and SYTO-61 (APC, 660/20 nm), Alexa Fluor 488 (FITC, 530/30 nm), and Hoechst 33342 (Pacific Blue, 450/50 nm)

## Software

1. FACSDiva 8.0.1 (BD Biosciences, https://www.bdbiosciences.com/en-au/products/software/instrument-software/bd-facsdiva-software)
2. FlowJo v10 (BD Biosciences, https://www.flowjo.com/solutions/flowjo)
3. Prism 9 (Dotmatics, GraphPad, https://www.graphpad.com/scientific-software/prism/)

## Procedure

All sample preparation is performed under sterile conditions. Centrifugation steps are at either 528× *g* for 5 min (5 acc/1 dec) (A3–B1) or 528× *g* for 90 s (9 acc/9 dec) for 96-well plates. Slow deceleration is used when centrifugation is performed in Falcon tubes to reduce the disturbance of the infected RBCs pellet.

- **Prepare malaria parasite cell culture**
  1. Maintain parasite-infected RBC culture at 5% hematocrit (percentage by volume of RBCs in culture) with O+ RBCs in CCM at 37 °C in a low oxygen environment (malaria mix). As an example, a 35 mm × 10 mm tissue culture dish would contain 250 μL of RBCs and 4.75 mL of CCM, yielding a 5% hematocrit culture. O+ RBCs are used as they are compatible with pooled sera from any blood type, avoiding RBC agglutination.
  2. Parasitemia (the percentage of infected RBCs relative to total RBCs) should be kept at ≤ 5% and the CCM replaced every 24–48 h. Monitor the parasitemia via thin blood smears and Giemsa staining.
    - Thin blood smears and Giemsa staining: Deposit 1–2 μL of RBCs at one end of a glass microscope slide. Use another glass slide held at a 45° angle to smear the cells across the first slide. Allow to air dry; then, fix the cells by immersing the slide in methanol for at least 5 s. Allow the slide to air dry and incubate the fixed slide in 10% Giemsa stain diluted in water for 5 min. Remove the slide from the stain and wash off excess stain with tap water. Blot the slide dry with paper towels; then, add immersion oil and visualize the cells with a 100× objective on a light microscope. Cell fixation and staining can be done in a non-sterile environment. Parasites will be stained dark purple and RBCs light purple. Use a physical cell counter to count ≥ 500 cells to determine the parasitemia, or percentage of cells infected with a parasite, prior to an experiment.
  3. Synchronize parasite cultures using the osmolyte D-sorbitol. Parasite-infected RBCs from 20 h post invasion (hpi) are susceptible to hypotonic lysis when incubated in a 5% solution of D-Sorbitol (Ginsburg et al., 1983; Counihan et al., 2017). This physiology is leveraged to synchronize cultures, as ring-stage parasites survive sorbitol treatment.
    Harvest the culture by centrifugation at 528× *g* for 5 min (5 acc/1 dec), aspirate the media, and resuspend the pellet in 5% D-sorbitol (w/v) to obtain a 5% hematocrit solution (the original volume of the culture). Incubate the D-sorbitol-treated culture in a water bath at 37 °C for 5–10 min. Harvest the sorbitol-treated culture, aspirate the supernatant, resuspend the pellet in CCM to 5% hematocrit, and return the culture to a fresh culturing dish or flask. One sorbitol synchronization will retain parasites that are ~0–20 hpi. Sorbitol-synchronize the parasites again 8 h later to narrow the age range to ~8–20 hpi. For the protocol outlined here, parasites must be synchronized to ~20–32 hpi (i.e., two synchronizations, 12 h apart).
  4. Transgenic parasites are maintained in the presence of selection reagent. To generate transgenic parasite cell lines, follow the detailed protocols and recipes outlined in Rug and Maier (2013). Our transgenic parasite cell lines were generated in a CS2 background, an isolate expressing the EMP1 variant VAR2CSA (Duffy et al., 2006). It is recommended to use the parent cell line as a positive control, as we do in this protocol.
    CS2 is a clonal parasite line that expresses *var2csa* as a dominant *var* gene transcript, allowing us to use an antibody for the encoded antigen (the EMP1 variant *var2csa*) to assess antigen trafficking to the host cell surface (Elliott et al., 2005; Duffy et al., 2006).
- **Dilute and aliquot cells**
  1. The infected RBC culture should be at 5% hematocrit with a parasitemia between 3% and 5%. Harvest mid-trophozoite stage cultures (~20–32 hpi) by centrifugation at 528× *g* for 5 min (5 acc/1 dec).
  2. Aspirate the spent media leaving the RBC pellet undisturbed. Resuspend the RBCs in 1% BSA/PBS and dilute as required to obtain a solution containing a 3%–5% parasitemia and 2%–4% hematocrit.
  3. Load 20 μL of diluted cells into a 96-well plate in duplicate or triplicate per condition and cell line. At a minimum, you will require a no-primary control (wells incubated with the BSA diluent and that receive the second and third antibody treatments), totalling ≥ 6 wells per cell line if performed in triplicate.
  4. Add 100 μL of wash buffer (1% BSA/PBS) to each well. Alternatively, add 100 µL of wash buffer prior to loading cells in step B3. We recommend a multichannel pipette for all subsequent washing steps.
  5. Centrifuge the plate at 528× *g* for 90 s (9 acc/9 dec) to pellet the cells, then aspirate the media using an aspirating tip. We find that the accuracy of aspiration is improved when a sterile pipette tip (no filter) is mounted on the end of the aspirator tip. Touch the tip to the wall of each well to aspirate the supernatant.
- **Cell staining**
*Note: All antisera are diluted in wash buffer (1% BSA/PBS).*
  1. Resuspend each well with 10 μL of either rabbit polyclonal anti-VAR2CSA antibody (1:100) or 1% BSA/PBS as a control and incubate for 30 min at 37 °C.
  2. Add 100 μL of wash buffer (1% BSA/PBS), centrifuge the plate, and aspirate the supernatants.
  3. Resuspend each well in 100 μL of wash buffer (1% BSA/PBS), centrifuge the plate, and aspirate the supernatants.
  4. Resuspend each well with 10 μL of mouse anti-rabbit IgG (1:100) and incubate for 30 min at 37 °C.
  5. Repeat steps C2 and C3 (wash steps).
  6. Resuspend each well with 10–20 μL of goat anti-mouse antibody conjugated to a fluorophore (1:100) and incubate for 30 min at 37 °C.
    - For cell lines expressing GFP, we use Alexa Fluor 647 goat anti-mouse IgG (H+L) and Hoechst 33342 DNA stain.
    - For cell lines not expressing GFP, we use Alexa Fluor 488 goat anti-mouse IgG (H+L) and Hoechst 33342 or SYTO-61 nucleic acid stain.
  7. Repeat steps C2 and C3.
  8. Incubate the cells in the DNA or nucleic acid stain.
    - For Hoechst 33342 staining, add 20 μL of stain (1:2,000) and incubate for 30 min at 37 °C. Wash cells twice (step C5), then wash the cells once in 100 μL of PBS alone. After this final wash, resuspend the cells in 200 μL of PBS (0.2%–0.4% hematocrit final), and then proceed with flow cytometry measurements. We use low hematocrit for these experiments to reduce the likelihood of capillary blockage during flow cytometry.
    - For SYTO-61 staining, incubate cells in 20 μL of SYTO-61 (5 μM in PBS) for 15 min at room temperature (Klonis et al., 2011). Add 180 μL of PBS to dilute the wells to 0.02% hematocrit and incubate the cells for 30 min at room temperature before being measured.
- **Flow cytometry using FACSDiva software**
  The steps below refer to a non-GFP-expressing cell line, labeled with Alexa Fluor 488 tertiary antibody and Hoechst 33342. Perform these steps for one well containing a double-stained positive sample (or well), e.g., the positive control, then apply the gating strategy to all wells in the plate.
  1. Use a side scatter height (SSC-H) vs. forward scatter area (FSC-A) plot to identify and gate the total RBC population (Figure 2A). Select this population.
  2. Then, use a forward scatter height (FSC-H) vs. forward scatter width (FSC-W) plot to gate singlet events (Figure 2B). Select this population.
  3. Plot Pacific blue height (DNA stain) vs. FITC height (fluorophore-conjugated tertiary antibody) (Figure 2C). Adjust the voltage of Pacific blue and FITC so that the Pacific blue-H +/- and FITC-H +/- populations can be clearly delineated. In our example plot (Figure 2C), the quadrant divide sits at approximately 10^3^. Once the voltages are optimized, apply these acquisition paraments to all wells in the plate and all biological repeats. As visualized in Figure 1, the Q4 population represents uninfected RBCs, the Q1 population is infected but does not have labeled EMP1 on the surface, and the Q2 population is both infected and presenting EMP1 on the surface.
*Note: If there is a substantial number of high-FITC events to the right of the main Q2 population, these are likely non-specific events. Consider increasing the volume of wash buffer during the wash steps or the number of washes after the final antisera incubation. Alternatively, use the secondary-only antibody controls as a guide for drawing a right boundary on the FITC+ populations.*
  4. Update the high throughput sampler (HTS) loader settings (Table 1).
    **Table 1.** Flow cytometer HTS loader settings

Sample flow rate (μL/s)	0.5
Sample volume (μL)	20
Mixing volume (μL)	100
Mixing speed (μL/s)	200
Number of mixes	4
Wash volume (μL)	800
  5. Collect a total of 50,000 events per well.
  6. Export all data as FCS-3 files.
  7. For instrument cleanup, flush the system sequentially with ≥ 400 μL of 0.1% (w/v) Triton X-100, ≥ 400 μL of 1% sodium hypochlorite, and ≥ 800 μL of ultra-pure water.
    **Figure 2.**
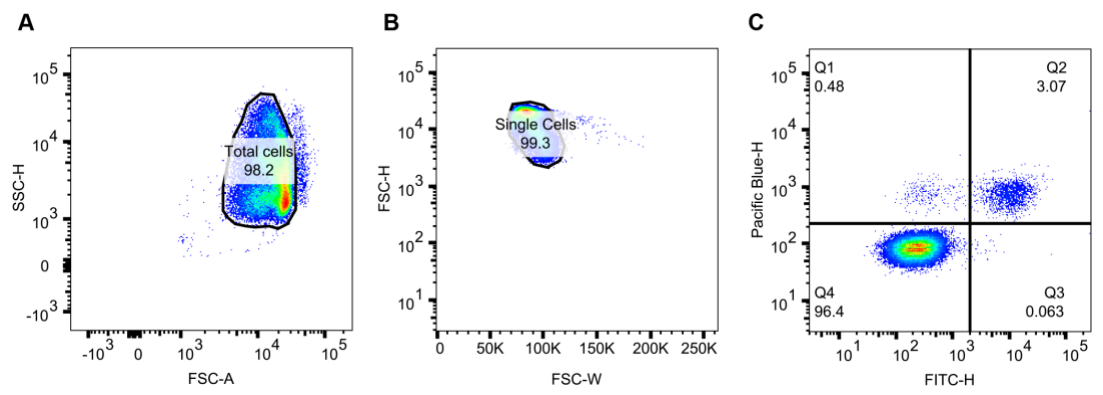
Flow cytometry analysis of a single well. Dots represent events detected. Blue to green to red hues indicate increasing population densities. (A) Side scatter height vs. forward scatter area to gate the major population, or *total cells* (infected and uninfected RBCs). (B) Doublet discrimination by plotting forward scatter height vs. forward scatter width and gating for the major population, or *single cells*. (C) Pacific blue height vs. FITC height to parse the *single cell* events into quartiles. Q2, the Pacific blue+/FITC+ population as a percentage of the single cell events, is used to compare the surface presentation of VAR2CSA between cell lines.

## Data analysis

1. Open FCS-3 files in FlowJo and apply the same gating strategy as outlined above (Figure 2A and 2B).
2. After delineating the total population into each quartile (Figure 2C), export the cell frequency of parent data for Q2 (i.e., the percentage of cells in Q2 compared to the single cell gate) for all wells.
3. Average the Q2 frequency across the technical repeats for each cell line and condition independently.
4. Subtract the Q2 averaged frequency of the no-primary controls from that of the experimental samples. This operation accounts for non-specific events in Q2.
5. Collect at least four biological repeats and perform the analysis above.
6. Using Prism 9 software, enter the biological replicate values in a column table where each column is a unique parasite cell line. Perform an unpaired *t*-test with Welch’s correction to determine the p-value.
7. Plot individual values as a scatterplot with bar (mean and standard deviation).

## Notes

Cell staining incubations were performed at 37 °C in standard atmospheric conditions. If practical, these incubations may be performed in malaria gas mix.

If live cell flow cytometry is not an option, the cells can be fixed after step C8. Fixation may be performed by incubating each sample with 20 μL of 4% Formaldehyde/0.0065% glutaraldehyde in PBS for 10 min, followed by three washes with 200 μL of PBS.

## Recipes

1. **Complete culture media (CCM)**
  Thaw then add the following reagents to 500 mL of RPMI 1640 medium with GlutaMAX and HEPES, store at 4 °C.
  25 mL of pooled human sera
  25 mL of 5% w/v Albumax II, dissolved in RPMI 1640 medium with GlutaMAX and HEPES
  5 mL of media solution (910 mM D-glucose, 0.45 mg/mL gentamicin)
  1 mL of 200 mM hypoxanthine

## References

[1] Beeson J. G., Mann E. J., Elliott S. R., Lema V. M., Tadesse E., Molyneux M. E., Brown G. V. and Rogerson S. J.(2004). Antibodies to variant surface antigens of *Plasmodium falciparum*-infected erythrocytes and adhesion inhibitory antibodies are associated with placental malaria and have overlapping and distinct targets. J Infect Dis 189(3): 540-551. 14745713 10.1086/381186 PMC2613478

[2] Boddey J. A., Carvalho T. G., Hodder A. N., Sargeant T. J., Sleebs B. E., Marapana D., Lopaticki S., Nebl T. and Cowman A. F.(2013). Role of plasmepsin V in export of diverse protein families from the *Plasmodium falciparum* exportome. Traffic 14(5): 532-550. 23387285 10.1111/tra.12053

[3] Carmo O. M. S., Shami G. J., Cox D., Liu B., Blanch A. J., Tiash S., Tilley L. and Dixon M. W. A.(2022). Deletion of the *Plasmodium falciparum* exported protein PTP7 leads to Maurer's clefts vesiculation, host cell remodeling defects, and loss of surface presentation of EMP1. PLoS Pathog 18(8): e1009882. 35930605 10.1371/journal.ppat.1009882 PMC9385048

[4] Cooke B. M., Buckingham D. W., Glenister F. K., Fernandez K. M., Bannister L. H., Marti M., Mohandas N. and Coppel R. L.(2006). A Maurer’s cleft-associated protein is essential for expression of the major malaria virulence antigen on the surface of infected red blood cells. J Cell Biol 172(6): 899-908. 16520384 10.1083/jcb.200509122 PMC2063733

[5] Counihan N. A., Chisholm S. A., Bullen H. E., Srivastava A., Sanders P. R., Jonsdottir T. K., Weiss G. E., Ghosh S., Crabb B. S., Creek D. J., .(2017). *Plasmodium falciparum* parasites deploy RhopH2 into the host erythrocyte to obtain nutrients, grow and replicate. Elife 6: e23217. 28252383 10.7554/eLife.23217 PMC5365316

[6] Duffy M. F., Maier A. G., Byrne T. J., Marty A. J., Elliott S. R., O’Neill M. T., Payne P. D., Rogerson S. J., Cowman A. F., Crabb B. S., .(2006). VAR2CSA is the principal ligand for chondroitin sulfate A in two allogeneic isolates of *Plasmodium falciparum*. Mol Biochem Parasitol 148(2): 117-124. 16631964 10.1016/j.molbiopara.2006.03.006

[7] Elliott S. R., Duffy M. F., Byrne T. J., Beeson J. G., Mann E. J., Wilson D. W., Rogerson S. J. and Brown G. V.(2005). Cross-reactive surface epitopes on chondroitin sulfate A-adherent *Plasmodium falciparum*-infected erythrocytes are associated with transcription of *var2csa*. Infect Immun 73(5): 2848-2856. 15845490 10.1128/IAI.73.5.2848-2856.2005 PMC1087379

[8] Ginsburg H., Krugliak M., Eidelman O. and Cabantchik Z. I.(1983). New permeability pathways induced in membranes of *Plasmodium falciparum* infected erythrocytes. Mol Biochem Parasitol 8(2): 177-190. 6348537 10.1016/0166-6851(83)90008-7

[9] Heiber A., Kruse F., Pick C., Gruring C., Flemming S., Oberli A., Schoeler H., Retzlaff S., Mesen-Ramirez P., Hiss J. A., .(2013). Identification of new PNEPs indicates a substantial non-PEXEL exportome and underpins common features in *Plasmodium falciparum* protein export. PLoS Pathog 9(8): e1003546. 23950716 10.1371/journal.ppat.1003546 PMC3738491

[10] Jensen A. R., Adams Y. and Hviid L.(2020). Cerebral *Plasmodium falciparum* malaria: The role of PfEMP1 in its pathogenesis and immunity, and PfEMP1-based vaccines to prevent it. Immunol Rev 293(1): 230-252. 31562653 10.1111/imr.12807 PMC6972667

[11] Klonis N., Crespo-Ortiz M. P., Bottova I., Abu-Bakar N., Kenny S., Rosenthal P. J. and Tilley L.(2011). Artemisinin activity against *Plasmodium falciparum* requires hemoglobin uptake and digestion. Proc Natl Acad Sci U S A 108(28): 11405-11410. 21709259 10.1073/pnas.1104063108 PMC3136263

[12] Kriek N., Tilley L., Horrocks P., Pinches R., Elford B. C., Ferguson D. J., Lingelbach K. and Newbold C. I.(2003). Characterization of the pathway for transport of the cytoadherence-mediating protein, PfEMP1, to the host cell surface in malaria parasite-infected erythrocytes. Mol Microbiol 50(4):1215-1227. 14622410 10.1046/j.1365-2958.2003.03784.x

[13] Marti M., Good R. T., Rug M., Knuepfer E. and Cowman A. F.(2004). Targeting malaria virulence and remodeling proteins to the host erythrocyte. Science 306(5703): 1930-1933. 15591202 10.1126/science.1102452

[14] McHugh E., Batinovic S., Hanssen E., McMillan P. J., Kenny S., Griffin M. D., Crawford S., Trenholme K. R., Gardiner D. L., Dixon M. W., .(2015). A repeat sequence domain of the ring-exported protein-1 of *Plasmodium falciparum* controls export machinery architecture and virulence protein trafficking. Mol Microbiol 98(6): 1101-1114. 26304012 10.1111/mmi.13201 PMC4987487

[15] McHugh E., Carmo O. M. S., Blanch A., Looker O., Liu B., Tiash S., Andrew D., Batinovic S., Low A. J. Y., Cho H. J., .(2020). Role of *Plasmodium falciparum* Protein GEXP07 in Maurer’s Cleft Morphology, Knob Architecture, and *P. falciparum* EMP1 Trafficking. mBio 11(2): e03320-19. 32184257 10.1128/mBio.03320-19 PMC7078486

[16] Reeder J. C., Cowman A. F., Davern K. M., Beeson J. G., Thompson J. K., Rogerson S. J. and Brown G. V.(1999). The adhesion of *Plasmodium falciparum*-infected erythrocytes to chondroitin sulfate A is mediated by *P. falciparum* erythrocyte membrane protein 1. Proc Natl Acad Sci U S A 96(9): 5198-5202. 10220443 10.1073/pnas.96.9.5198 PMC21841

[17] Rug M. and Maier A. G.(2013). Transfection of *Plasmodium falciparum*. Methods Mol Biol 923: 75-98. 22990772 10.1007/978-1-62703-026-7_6

[18] Sahu P. K., Duffy F. J., Dankwa S., Vishnyakova M., Majhi M., Pirpamer L., Vigdorovich V., Bage J., Maharana S., Mandala W., .(2021). Determinants of brain swelling in pediatric and adult cerebral malaria. JCI Insight 6(18): e145823. 34549725 10.1172/jci.insight.145823 PMC8492338

[19] Sargeant T. J., Marti M., Caler E., Carlton J. M., Simpson K., Speed T. P. and Cowman A. F.(2006). Lineage-specific expansion of proteins exported to erythrocytes in malaria parasites. Genome Biol 7(2): R12. 16507167 10.1186/gb-2006-7-2-r12 PMC1431722

[20] Smith J. D., Chitnis C. E., Craig A. G., Roberts D. J., Hudson-Taylor D. E., Peterson D. S., Pinches R., Newbold C. I. and Miller L. H.(1995). Switches in expression of *Plasmodium falciparum* var genes correlate with changes in antigenic and cytoadherent phenotypes of infected erythrocytes. Cell 82(1): 101-110. 7606775 10.1016/0092-8674(95)90056-x PMC3730239

[21] Storm J. and Craig A. G.(2014). Pathogenesis of cerebral malaria--inflammation and cytoadherence. Front Cell Infect Microbiol 4: 100. 25120958 10.3389/fcimb.2014.00100 PMC4114466

[22] Weiss D. J., Lucas T. C. D., Nguyen M., Nandi A. K., Bisanzio D., Battle K. E., Cameron E., Twohig K. A., Pfeffer D. A., Rozier J. A., .(2019). Mapping the global prevalence, incidence, and mortality of *Plasmodium falciparum*, 2000-17: a spatial and temporal modelling study. Lancet 394(10195): 322-331. 31229234 10.1016/S0140-6736(19)31097-9 PMC6675740

